# Risk of Developing Depressive Disorders following Hepatocellular Carcinoma: A Nationwide Population-Based Study

**DOI:** 10.1371/journal.pone.0135417

**Published:** 2015-08-21

**Authors:** Chun-Hung Chang, Shaw-Ji Chen, Chieh-Yu Liu

**Affiliations:** 1 Department of Psychiatry, China Medical University Hospital, Taichung, Taiwan; 2 Institute of Clinical Medicine, China Medical University, Taichung, Taiwan; 3 Department of Psychiatry, Mackay Memorial Hospital Taitung Branch, Taitung, Taiwan; 4 Mackay Junior College of Medicine, Nursing, and Management, Taipei, Taiwan; 5 Institute of Medical Sciences, Tzu Chi University, Hualien, Taiwan; 6 Institute of Nursing-Midwifery, National Taipei University of Nursing and Health Sciences, Taipei, Taiwan; University of Modena & Reggio Emilia, ITALY

## Abstract

**Background & Aims:**

To evaluate the risk of depressive disorders among patients with Hepatocellular Carcinoma (HCC) using the National Health Insurance Research Database (NHIRD) in Taiwan.

**Methods:**

We conducted a retrospective study of a newly diagnosed HCC cohort of 55,973 participants who were selected from the NHIRD. Patients were observed for a maximum of 6 years to determine the rates of newly onset depressive disorders, and Cox regression was used to identify the risk factors associated with depressive disorders in HCC patients.

**Results:**

Of the total 55,973 HCC patients, 1,041 patients (1.86%) were diagnosed with depressive disorders during a mean (SD) follow-up period of 1.1 (1.2) years. The Cox multivariate proportional hazards analysis showed that age of 40–59 (HR 1.376, 95% CI 1.049–1.805, p = 0.021), age of 60–79 (HR 1.341, 95% CI 1.025–1.753, p = 0.032), women (HR 1.474 95% CI 1.301–1.669, p < 0.001), metastasis (HR 1.916, 95% CI 1.243–2.953, p = 0.003), and HCV (HR 1.445, 95% CI 1.231–1.697, p < 0.001) were independent risk factors for developing depressive disorders.

**Conclusions:**

Our study indicated a subsequent risk of depressive disorders in patients with HCC, and the risk increased for those with female gender, aged 40 to 59, aged 60 to 79, with metastasis, or with HCV. Psychological evaluation and support are two critical issues in these HCC patients with the risk factors.

## Introduction

Hepatocellular Carcinoma (HCC) is the fifth most common cancer worldwide, and the annual global incidence is about 1 million cases with a male to female ratio of approximately 4:1,and higher incidence rates in Asian countries where infection with hepatitis B virus (HBV) is endemic [1, 2]. Surgery is considered the standard curative treatment, but nonsurgical therapies can prolong the survival period and palliate symptoms [3–5]. Considerable studies have demonstrated the high prevalence of symptoms of depression in cancer patients [6], and depression as a predictor of cancer progression and mortality[7]. But few studies investigate the relationship between HCC and depression. Four studies [8–11] and one review [12] have revealed health-related quality of life (HRQOL) was correlated negatively with depression. Besides, two studies have shown HCC patients treated with hepatic resection had better HRQOL and were less depressed than patients treated by hepatic arterial infusion [13, 14].

However, these studies were designed to evaluate HRQOL, not designed to address the risk of depressive disorders, which are clinical conditions with depressed mood, experiencing a loss of energy and interest, feelings of guilt, difficulty in concentrating, loss of appetite, and thoughts of death or suicide [15]. Moreover, these studies are based on screening instruments rather than clinical diagnosis by physicians [12]. Therefore, the risk of depressive disorders following HCC remains unknown. In summary, according to the current published cancer-related papers, lots of published papers focused on the cancer-induced fatigue, quality of life or some mental disorders measured by questionnaires, and relatively less studies focused on the physician-confirmed depressive disorders in HCC patients. This study was aimed to investigate the subsequent risk of depressive disorders in HCC patients, using the population-based retrospective cohort derived from the National Health Insurance Research Database (NHIRD) in Taiwan.

## Methods

### Data sources

In Taiwan, the National Health Insurance (NHI) program was initiated by the Taiwanese government since March 1, 1995 and covered over 98.29% of residents [16]. The NHI research database (NHIRD) contains comprehensive information including prescription details, clinical visits, and diagnostic codes. In the NHIRD, the International Classification of Diseases, Ninth revision, Clinical Modification (ICD-9-CM) and Procedure Coding System (ICD-9-PCS) are used to define the diagnostic and procedure codes.

### Ethics statement

This study was approved by the Institutional Review Board of the China Medical University Hospital (CMUH103-REC3-077). Written consent from study patients was not obtained because all information potentially identifying any individual patient was encrypted. The data regulations of the patients’ confidentiality were guaranteed by the Bureau of NHI and Institutional Review Board of China Medical University Hospital.

### Study population

**Fig 1:**
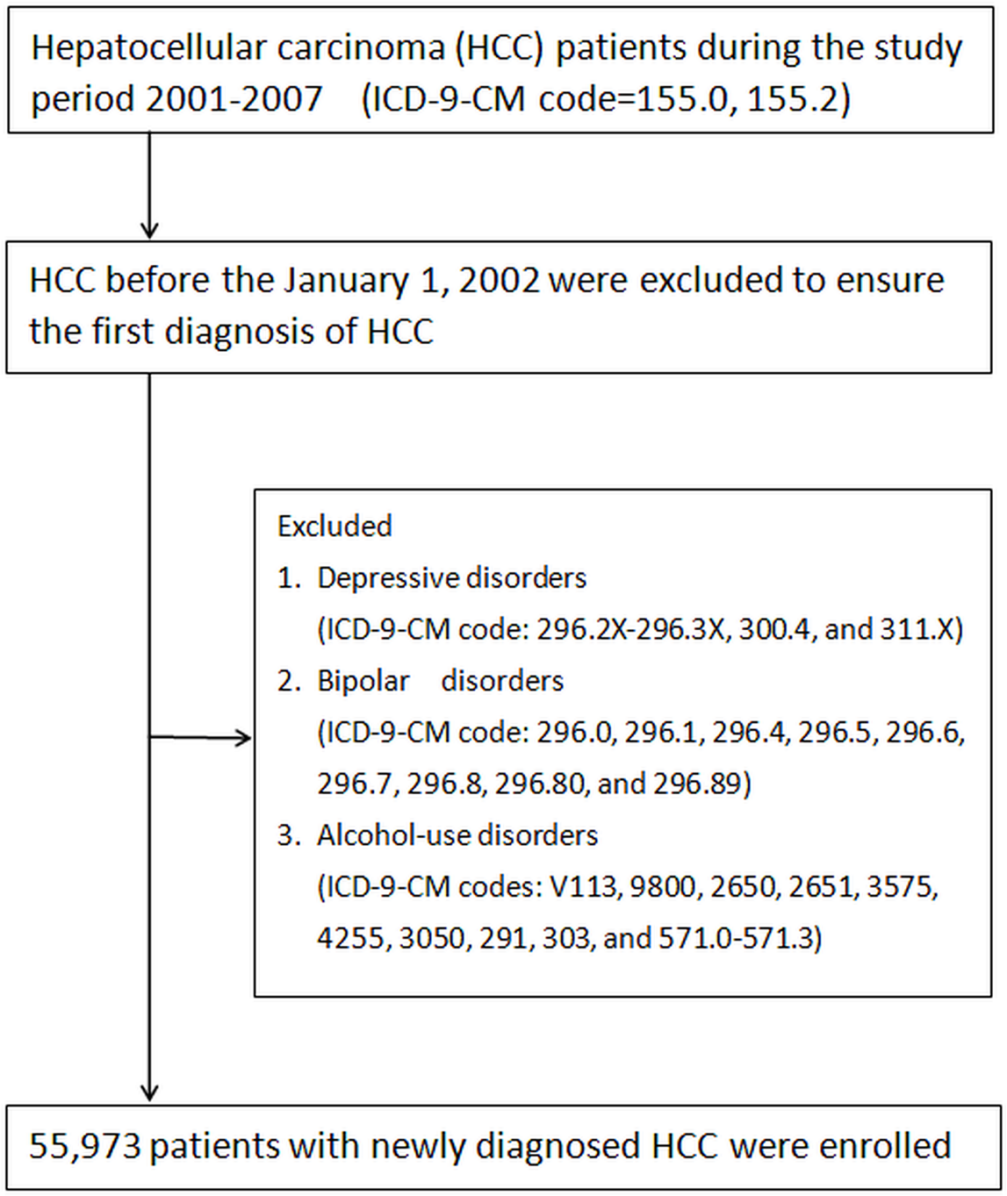
Selection of study patients.

We identified all patients with a primary diagnosis of HCC (ICD-9-CM code = 155.0, 155.2) for the first time between January 1, 2001, and December 31, 2007 from NHIRD. Those with a documented HCC before the January 1, 2002 were excluded to ensure the first diagnosis of HCC. We excluded subjects diagnosed with depressive disorders (ICD-9-CM code: 296.2X-296.3X, 300.4, and 311.X) before enrollment to identify patients with the newly-onset depressive disorders. In Taiwan, a diagnosis of depressive disorders was made according to ICD-9 CM code and Diagnostic and Statistical Manual of Mental Disorders (DSM)–IV [17] by board-certified psychiatrists and physicians. In this study, only those having at least three consecutive corresponding diagnoses were designated as having depressive disorder for better diagnostic validity. For preventing confounding effects, we followed a flowchart of a previous study [18] and excluded patients with bipolar disorders (ICD-9-CM code: 296.0, 296.1, 296.4, 296.5, 296.6, 296.7, 296.8, 296.80, and 296.89), or alcohol-use disorders (ICD-9-CM codes: V113, 9800, 2650, 2651, 3575, 4255, 3050, 291, 303, and 571.0–571.3) because higher rate of impaired liver function due to mood stabilizers such as valproate, carbamazepine, or alcohol use [15]. All patients with HCC were observed until diagnosed with depressive disorders according to ICD-9-CM codes (ICD-9-CM code: 296.2X-296.3X, 300.4, and 311.X), death, or withdrawal from the NHI system, or December 31, 2007. (Fig 1)

### Covariate assessment

Because the chance of HCC recurrence can be confounded by competing risk of mortality, we identified co-morbidities that may be associated with mortality based on diagnostic codes prior to the outcome of interest. All diseases included in the Charlson Comorbidity Index were analyzed [19]. Comorbidities, listed by ICD-9code, included hepatitis B virus (HBV) infection (ICD-9 codes 070.2, 070.3, and V02.61), hepatitis C virus (HCV) infection (ICD-9codes 070.41, 070.44, 070.51, 070.54, and V02.62), acute coronary syndrome (410–414), cerebrovascular accident (430–438), chronic obstructive pulmonary disease (COPD) (490–496), diabetes mellitus (250), liver cirrhosis (571.5), liver failure (570), renal failure (584–586), hypertension (401–405), hyperlipidemia (272.0–272.2), and peptic ulcer disease (531–534). Metastasis was defined as secondary malignant neoplasm of liver (code 197.7).

### Statistical analyses

The dependent variable was the incidence of depressive disorders. Each patient was followed for a maximum of 6 years since 2001 January. The independent sample t-test was used for the difference comparison of age, follow-up years and CCI scores between HCC patients with- and without depressive disorder. The Chi-square test was used for the distribution comparison of categorical variables, including age groups, sex, and major existing diseases. The Kaplan–Meier method was employed for estimation of cumulative incidence between male and female. We further used Cox proportional hazard model to identify risk factors for depressive disorders. The covariate adjustments which we took into account were age, sex, and comorbidities. Due to the limitations of personal privacy protection, the NHIRD do not release the personal information of ethnicity and socioeconomic status, which were considered one study limitation in this study. The database software of MY Structured Query Language (MySQL) was adopted for extraction, linkage, and processing of the study database. All statistical analyses were performed using IBM SPSS statistical software (version 19.0 for Windows; IBM Corp., New York, NY, USA), and two-tailed p-value < 0.05 was considered to be statistically significant.

## Results

### Clinical characteristics of the study population

During the study period, a total of 55,973 patients with newly diagnosed HCC were enrolled. The patients had a mean±SD age of 61.3±13.6 years. The most common underlying diseases were cirrhosis (36.5%), peptic ulcer diseases (16.6%), and HCV (15.4%). The mean follow-up period for the group was 3.0±1.7 years (Table 1).

**Table 1:** Basic Profile of Patients with HCC (n = 55,973).

Variable	Number	(%)
Age, y	61.3	(13.6)
20–39	4,119	(7.4)
40–59	20,231	(36.1)
60–79	27,837	(49.7)
> = 80	3,876	(6.8)
Sex		
Men	37,790	(67.5)
Women	18,183	(32.5)
Follow-up, y		
Mean (SD)	3.0 (1.7)	
Major coexisting diseases		
Cirrhosis	20,419	(36.5)
Peptic ulcer diseases	9,307	(16.6)
HCV	8,640	(15.4)
Diabetes	7,347	(13.1)
Hypertension	6,914	(12.4)
COPD	1,966	(3.5)
HBV	1,287	(2.3)
Renal failure	726	(1.3)
Acute coronary syndrome	571	(1.0)
Hyperlipidemia	515	(0.9)
Cerebral vascular disease	444	(0.8)
Metastasis	588	(1.1)
Charison score	2.5 (3.4)	

Abbreviations: HCC, hepatocellular carcinoma; SD, standard deviation; HCV, hepatitis C virus; COPD, chronic obstructive pulmonary disease; HBV, hepatitis B virus.

### Incidence rates of depressive disorders

**Fig 2:**
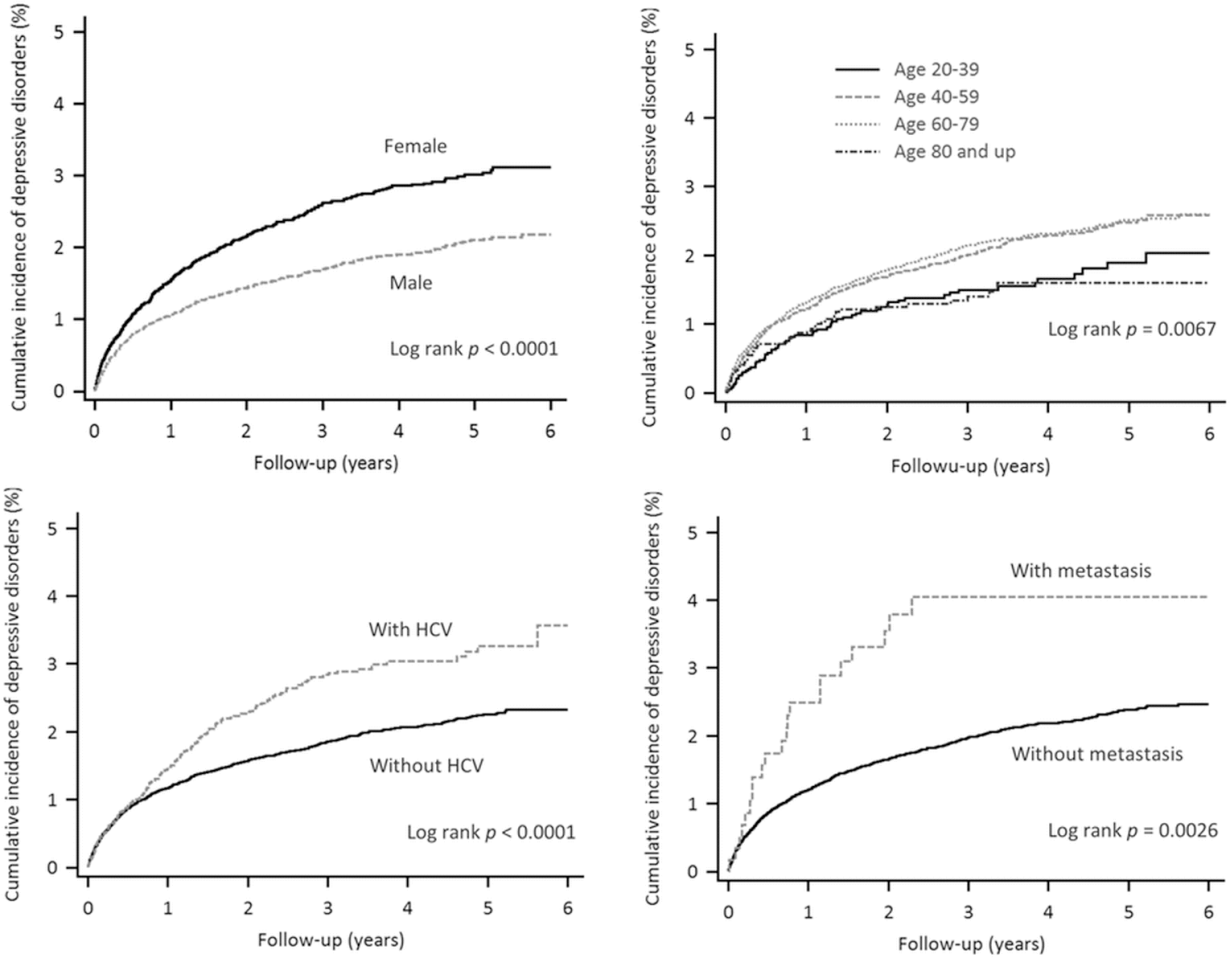
Cumulative incidence of depressive disorders in HCC patients with female or male gender, in four age groups, with or without HCV, and with or without metastasis.

Of the total 55,973 patients, 1,041 patients (1.86%) were diagnosed with depressive disorders during 6-year observation. Among those with depressive disorders, 607 (58.3%) were male and 434 (41.7%) were female, with 61 (5.9%) 20 to 39 years of age, 385 (37.0%) 40 to 59 years of age, 547 (52.5%) 60 to 79 years of age, and 48 (4.6%) > = 80 years of age (Table 2). Kaplan–Meier estimate of the cumulative incidence of depressive disorders in the HCC patients is 2.5% (3.1% for women and 2.2% for men respectively shown in Fig 2. The cumulative incidence of depressive disorders was significantly higher for the female patients, 40 to 59 years of age, with HCV, and with metastasis (3.1%, 2.6%, 3.6%, and 4.0% respectively).

**Table 2:** Comparisons in demographic characteristics in HCC survivors with and without depressive disorders (n = 55,973).

	Depressive disorders				
	No (n = 54,932)		Yes (n = 1,041)		
	n	(%)	n	(%)	*p*-value
Age, mean±SD*, y	61.3±13.6		61.1±12.5		0.713
20–39 y	4,058	(7.4)	61	(5.9)	0.006
40–59 y	19,846	(36.1)	385	(37.0)	
60–79 y	27,290	(49.7)	547	(52.5)	
≧ 80 y	3,738	(6.8)	48	(4.6)	
Sex					
Men	37183	(67.7)	607	(58.3)	<0.001
Women	17749	(32.3)	434	(41.7)	
Follow-up, mean±SD, y	3.1±1.7		1.1±1.2		<0.001
Major coexisting diseases					
Cirrhosis	20,078	(36.6)	341	(32.8)	0.012
Peptic ulcer diseases	9,121	(16.6)	186	(17.9)	0.278
HCV	8,436	(15.4)	204	(19.6)	<0.001
Diabetes	7230	(13.2)	117	(11.2)	0.069
Hypertension	6777	(12.3)	137	(13.2)	0.424
COPD	1921	(3.5)	45	(4.3)	0.152
HBV	1264	(2.3)	23	(2.2)	0.845
Renal failure	715	(1.3)	11	(1.1)	0.489
Acute coronary syndrome	559	(1.0)	12	(1.2)	0.667
Hyperlipidemia	509	(0.9)	6	(0.6)	0.241
Cerebral vascular disease	433	(0.8)	11	(1.1)	0.333
Metastasis	567	(1.0)	21	(2.0)	0.002
Charison score, mean±SD	2.5±3.4		2.5±3.3		0.903

Chi-square test, and *t-test comparing subjects with depressive disorders and non- depressive disorders. Abbreviations: HCC, hepatocellular carcinoma; SD, standard deviation; HCV, hepatitis C virus; COPD, chronic obstructive pulmonary disease; HBV, hepatitis B virus.

### Risk factors for depressive disorders in the cohort

The Cox univariate proportional hazards analysis showed that patients with female gender, aged 40 to 59, metastasis, and HCV had higher risk of depressive disorders. In the Cox multivariate proportional hazards analysis, age of 40–59 (HR 1.376, 95% CI 1.049–1.805, p = 0.021), age of 60–79 (HR 1.341, 95% CI 1.025–1.753, p = 0.032), women (HR 1.474 95% CI 1.301–1.669, p < 0.001), metastasis (HR 1.916, 95% CI 1.243–2.953, p = 0.003), and HCV (HR 1.445, 95% CI 1.231–1.697, p < 0.001) were independent risk factors for developing depressive disorders (Table 3).

**Table 3:** Univariate and multivariate survival analysis for factors associated with depressive disorders.

Variables	Univariate analysis			Multivariate analysis		
	HR	95% CI of HR	p-value	aHR	95% CI of aHR	p-value
Age						
20–39(ref)	1.000					
40–59	1.348	1.029–1.766	0.030	1.376	1.049–1.805	0.021
60–79	1.389	1.066–1.810	0.015	1.341	1.025–1.753	0.032
> = 80	0.933	0.639–1.362	0.719			
Sex						
Men	1.000					
Women	1.217	1.144–1.294	<0.001	1.474	1.301–1.669	<0.001
Major coexisting diseases						
Cirrhosis	0.852	0.749–0.970	0.015	0.799	0.699–0.913	0.001
Peptic ulcer diseases	1.088	0.992–1.275	0.296			
HCV	1.439	1.234–1.677	<0.001	1.445	1.231–1.697	<0.001
Diabetes	0.870	0.718–1.055	0.157			
Hypertension	1.136	0.949–1.360	0.163			
COPD	1.272	0.944–1.715	0.114			
HBV	1.122	0.742–1.696	0.586			
Renal failure	0.829	0.458–1.502	0.536			
Acute coronary syndrome	1.140	0.645–2.014	0.652			
Hyperlipidemia	0.638	0.286–1.423	0.272			
Cerebral vascular disease	1.343	0.741–2.432	0.331			
Metastasis	1.921	1.247–2.960	0.003	1.916	1.243–2.953	0.003

Abbreviations: HCC, hepatocellular carcinoma; SD, standard deviation; HCV, hepatitis C virus; COPD, chronic obstructive pulmonary disease; HBV, hepatitis B virus; HR, hazard ratio; aHR, adjusted hazard ratio

## Discussion

To the best of current knowledge, the present study is the first study to analyze subsequent risk of depressive disorders in patients with HCC. The results of this nationwide, population-representative cohort study shows that (1) the incidence rate of depressive disorders in HCC patients was 2.5%; (2) the risk of depressive disorders was higher when patients were at 40 to 59 years of age, 60 to 79 years of age, the female sex, with metastasis, or with HCV.

In Taiwan, one cancer patient can freely consult psychiatrists without referral. In most Taiwanese situations, cancer patients are usually found with depressive disorder by their caregivers, therefore, the caregivers would arrange the psychiatrist’s visits for cancer patients. The study sample covered nearly the whole population and all HCC patients during the study period. Therefore, the study database may have very small risk of so-called “selection bias” in the present study. However, patients with minor depression disorder which may not be found by caregivers would not consult psychiatrists, so that we still may underestimate the incidence if using broader definition of depressive disorder. In the present study, the incidence of depressive disorders of our study was 6.13 per 1000 person-year, which was higher than the incidence of general population in Chien et al.’s survey in Taiwan (1.89 per 1000 person-year to 2.58 per 1000 person-year) [20], compared to the annual rates ranged from 0.8 cases per 100 adults in Taiwan to 5.8 cases per 100 adults in New Zealand [21]. Previous studies have shown higher depression scores in HCC subjects, who were evaluated by screen instruments like the health-related quality of life (HRQOL), or the Hamilton Depression Rating Scale (HAM-D) [12]. In a study of 24 HCC patients, 22 cirrhotic patients, and 20 control subjects, Hamilton Depression Rating Scale (HAM-D) HAM-D scores in HCC patients were higher than in healthy subjects (8.7 ± 4.4 vs 4.9 ± 2.9, p< 0.001) [22]. However, a retrospective case-control study reported that no association is detected between depression and risk of hepatocellular carcinoma in older people in Taiwan [23]. Besides, another cross-sectional study showed that neuropsychological symptoms were unrelated to cirrhosis stage and hepatocellular carcinoma [24]. In comparison with these two previous studies, this study used a national-wide database covering general population, including all age groups. Therefore, the results revealed in this study are believed more generalizable and may contribute more evidences for clinicians.

Our findings also showed that the subsequent risk of depressive disorders increased in HCC patients aged 40 to 59 years, or 60 to 79 years compared with aged 20 to 39 years. The onset age of depressive disorders in HCC survivors is late than in general population, whose mean age of onset for major depressive disorder is about 40 years, with 50 percent of all patients having an onset between the ages of 20 and 50 [15]. This may be resulted from (1) cancer is a risk factor for developing depression [25–29], and (2) HCC commonly occurs after the age of 40 years and reaches a peak at approximately 70 years of age [30].

Our large-scale study identified women as having higher risks of subsequent depressive disorders following HCC diagnosis. This might reflect a general finding that depression is more prevalent in women than in men, twofold greater prevalence of major depressive disorder in women than in men [31]. Hormonal differences, the effects of childbirth, differing psychosocial stressors for women and for men, and behavioral models of learned helplessness may be involved in the reasons for the difference [15].

Moreover, we observed an increased risk of depressive disorders among HCC survivors with HCV. One study has shown that HCV is one of predictors for depression because a total of 29.7% of 4,781 patients with HCV were depressed on the Patient Health Questionnaire-8 (PHQ-8) scale (scores > = 10) during 5-year follow-up[32]. Furthermore, a meta-analysis reported one in four chronic hepatitis C patients who start interferon and ribavirin treatment will develop an induced major depressive episode [33]. We also found that metastasis was an independent risk for depressive disorders, and agreed with previous study. Patients with advanced cancer have higher prevalence rate of major depression than general population (5%–26% vs 1.5–19.0%) [21, 28]. However, patients with cirrhosis in our study had lower risk. This finding was in contrast with previous studies [24, 34, 35]. Perng et al. reported that non-alcoholic cirrhotic patients had higher risk to develop depressive disorders than patients without cirrhosis (incidence risk ratio 1.76, 95% CI, 1.57–1.98, P<0.001) [18]. Further studies will need to investigate the subsequent depression in HCC patients with non-alcoholic cirrhosis.

This study had some limitations. First, the National Health Insurance Research Database did not supply data including cancer staging, genetic or environmental factors, all of the potential confounders which may be associated with the risk of depressive disorders. However, we adopted a comprehensive national database to lower the sample bias. Second, the patients in this study were Chinese, and, thus, the results of this study might not be generalizable to other ethnic populations. However, the East Asian population is at higher risk of HCC than other ethnic populations [30]; therefore, we used the large population-representative HCC cohort to evaluate the risk of depressive disorders. Third, this study might have underestimated HCC patients with depressive disorders because only those patients who received medical care were enrolled. Although the case definition was more conservative, our findings showed the incidence of depressive disorders in HCC patients is still significantly higher than that in general population. If we used a broader definition of depressive disorders in HCC patients, the actual incidence is believed to be even higher.

In conclusion, this study indicated a subsequent risk of depressive disorders in patients with HCC, and the risk increased for those with female gender, 40 to 59 years of age, 60 to 79 years of age, metastasis, or HCV. The HCC patients with these risk factors need more psychological evaluation and treatment. Further studies would be required to clarify the possible mechanisms of this association between depressive disorders and HCC.
